# Correction: Structure evolution, amorphization and nucleation studies of carbon-lean to -rich SiBCN powder blends prepared by mechanical alloying

**DOI:** 10.1039/c8ra90088d

**Published:** 2018-11-20

**Authors:** Daxin Li, Zhihua Yang, Dechang Jia, Shengjin Wang, Xiaoming Duan, Bin Liang, Qishuai Zhu, Yu Zhou

**Affiliations:** Institute for Advanced Ceramics, Harbin Institute of Technology Harbin 150001 China Zhyang@hit.edu.cn

## Abstract

Correction for ‘Structure evolution, amorphization and nucleation studies of carbon-lean to -rich SiBCN powder blends prepared by mechanical alloying’ by Daxin Li *et al.*, *RSC Adv.*, 2016, **6**, 48255–48271.

The authors regret that Fig. 13 was displayed incorrectly in the original article. Due to a data processing error, partially repetitive data was displayed for the entry for 10 h. The correct version of Fig. 13 is shown below.

**Fig. 13:**
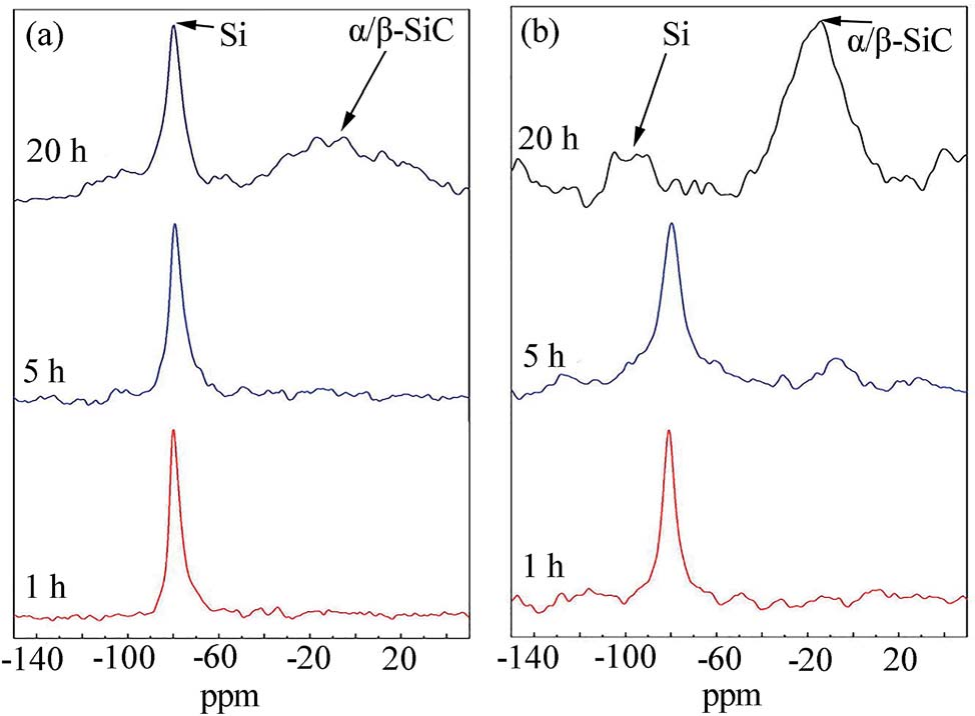
Solid-state ^29^Si NMR spectra of carbon-lean C2 (a) and carbon-rich C9 (b) powder blends subjected to different hours of milling.

The Royal Society of Chemistry apologises for these errors and any consequent inconvenience to authors and readers.

## Supplementary Material

